# Point-of-Care Monitoring of Respiratory Diseases Using Lateral Flow Assay and CMOS Camera Reader

**DOI:** 10.1109/JTEHM.2022.3193575

**Published:** 2022-08-10

**Authors:** Shasidran Raj, Darragh McCafferty, Gennady Lubrasky, Sarah Johnston, Kerry-Louise Skillen, James McLaughlin

**Affiliations:** Connected Health Innovation Centre, NIBECUlster University 2596 Belfast BT37 0QB U.K; ProAxsis Belfast BT3 9DT Northern Ireland; Eastern Corridor Medical Engineering Centre, NIBECUlster University 2596 Belfast BT37 0QB U.K

**Keywords:** Respiratory illness, point-of-care, lateral flow assays, neutrophil elastase, image analysis, CMOS camera

## Abstract

Objective: Respiratory disease is a rapidly growing global health issue that impacts the quality of living of tens of millions of people around the world. Neutrophil elastase (NE) represents a key inflammatory biomarker and has previously been demonstrated to have the capability of predicting exacerbation risk related to respiratory diseases. This paper utilises a low-cost Point of Care (PoC) approach using Lateral Flow Assays (LFAs) to provide quantitative measurement of active NE in a patient’s sputum. Methods and procedures: The main aim of this study is to develop a quantitative platform using a Complementary Metal-Oxide-Semiconductor (CMOS) to image the LFAs and with an adaptable image analysis algorithm to measure a target biomarker concentration. This result could be used to monitor a patient’s health and quality of living. In the paper, NE is used as the target biomarker to determine if the patient is suffering from a high risk of exacerbations. Results: The results presented in the paper indicate the CMOS reader approach is promising for rapid and low-cost PoC devices, with the current system able to provide quantitative trends of NE concentrations as low as 100 ng/ml and is comparable to a research-based laboratory lateral flow reader. Conclusion: The image analysis algorithm used in the CMOS reader can estimate the minimum NE concentration of 250 ng/ml to indicate the high-risk category for exacerbations from respiratory illnesses with the same accuracy as expensive a research-based laboratory reader but by using low-cost components and onboard image analysis. Clinical impact: The image analysis algorithm is evaluated to analyse LFAs with NE biomarker to determine the patient in a high-risk category for exacerbations. The device communicates the analysis result to medical professionals for daily historical logging for daily health monitoring without regular hospital appointments. The low-cost approach of the proposed system and image analysis approach can be adapted to analyse different biomarkers for other health concerns including multiplex LFAs without additional hardware in the reader design.

## I. Introduction

This paper aims to present the development and evaluation of a quantitative Lateral Flow Assays (LFAs) platform by utilising Complementary Metal-Oxide-Semiconductor (CMOS) to take images of the Lateral Flow Assays. The design and image analysis approach taken in the development of the CMOS reader to provide a quantitative analysis of NEATstik® LFAs used to estimate the concentrations of neutrophil elastase (NE) related to respiratory illness. The results aim to show the effectiveness, accuracy and adaptability of the approach where simple and low-cost components are used in the reader design but with the aid of powerful and adaptable onboard image analysis techniques to match the capabilities of a laboratory diagnostic tool. The CMOS reader design also implements the Internet of Things (IoT) for a Point-of-Care (PoC) approach.

An LFA is a simple, user-friendly and low-cost rapid test device used to identify the presence of a target substance from small quantities of patient biological samples [1], [2] attributed to different medical health conditions. Multiplex LFAs can also be designed to measure concentrations of multiple target substances simultaneously [3].

Visual observations of the LFAs can provide a semi-quantitative measurement of the concentration of the target substance, allowing for self-diagnosis and monitoring throughout the treatment. Modern LFA analysis systems can provide a more quantitative estimate of the concentration level of the target substance by incorporating a suitable diagnostic tool to analyse the LFA to improve the sensitivity and accuracy of the test result. Laboratory-based analysis tools can be sensitive with high accuracy but can also be expensive, not user-friendly and difficult to use in a PoC application. Alternative devices could use simple and low-cost light-sensitive measurement [1] or a mobile phone to take and analyse an image [2]. However, these methods would be sensitive to the mechanical alignment of the LFA with the sensors and external light conditions. These disadvantages would impact the sensitivity of LFA analysis results and hence introduce unwanted errors in estimating the target substance’s concentration. Most commercially available low-cost LFA readers are mechanically designed to analyse specific types of LFA strips but are not adaptable to analyse other types of LFAs or multiplex LFAs without changes to the reader design or hardware.

The advantage of the proposed CMOS reader system is the complexity of the system is shifted to the image analysis algorithm. This allows the reader to be more adaptable in analysing multiple types of LFAs for different medical conditions, including multiplex LFA strips with multiple biomarkers simultaneously. The analysis algorithm can also be constantly updated to improve the sensitivity of the analysis and also implement previous work with the CMOS camera images with the application of neural networks to analyse the LFA strips [4]. These analysis methods can be added to the images analysis algorithm presented in this paper, both onboard or on the cloud-based analysis platforms via software updates without requiring changes to the current hardware of the reader. The reader design can be kept simple and low-cost while still controlling the lighting conditions in the case when imaging the LFAs, unlike mobile phone analysis tools. Any LFA misalignments do not impact the image quality and can be corrected in post-processing by the image analysis algorithm.

Using IoT systems, the device can upload the image and results to a mobile phone or web server for historical logging and monitoring of the patient’s health. This approach allows patients to take regular LFA tests from home and the results can be uploaded for medical professionals to access and monitor the patient’s health without requiring the patient to regularly attend a clinic or hospital.

## II. Background

Respiratory diseases constitute one of the major public health challenges in the world today. Conditions such as Chronic Obstructive Pulmonary Disease (COPD) and Bronchiectasis represent the common causes of morbidity and mortality within the global population [5], [6]. The World Health Organisation (WHO) estimates that 65 million people suffer from COPD, of which 3 million prematurely lose their lives each year, making it the $3^{rd}$ leading cause of death worldwide [7] and the prevalence of this disease has increased by nearly 50% between 1990 and 2017 [8].

Key drivers of respiratory disease progression are acute exacerbations, defined as the sustained worsening of symptoms, such as sputum volume, dyspnea and coughing. Frequent exacerbators typically experience poor quality of life, and an increased risk of mortality [9], [10]. The biggest challenge with tackling these diseases is the proper diagnosis and monitoring of these diseases. In most cases, monitoring a patient’s health is not possible outside a hospital environment and the patient’s health may deteriorate. Proper monitoring could allow doctors and clinicians to provide more suitable care for the patients and improve their quality of living.

### A. Using Neutrophil Elastase to Monitor Respiratory Diseases

NEATstik® is a CE registered PoC device that provides a qualitative measurement of active NE in sputum. The process of using NEATstik® to measure NE is similar to other LFA used in several different PoC implementations [1]. Although the existing iteration may have a role to play in routine patient monitoring, a quantitative version would provide greater objectivity during use. Moreover, these tests could facilitate the delineation of key disease thresholds, enabling preemptive medical intervention and thereby improving the standard of care received by patients with respiratory disease.

Respiratory physicians can provide improved disease management by searching for and monitoring the presence of exacerbation biomarkers, specifically those that exhibit diagnostic value. A recent bronchiectasis study by Shoemark *et al.*, highlighted the potential use of active NE to assist in the prediction of future exacerbation risks [11]. NE belongs to the chymotrypsin superfamily of serine proteases and represents a vital component of the innate immune response against invading pathogens, therefore contributing directly to the destruction of phagocytosed bacteria, and the inflammatory process via proteolytic activation of various cytokines and chemokines [12]. Therefore NE has been established as a biomarker of disease severity in several inflammatory respiratory diseases, including cystic fibrosis and bronchiectasis [13], [14].

**FIGURE 1.**
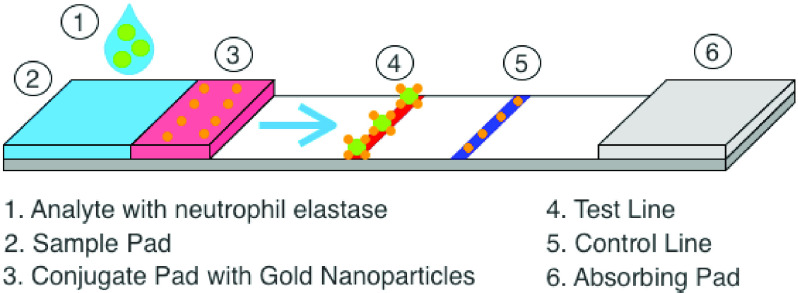
Illustration of NEATstik® device used to analyse a patient’s sputum to determine the NE concentration.

Figure 1 illustrates how a NEATstik® LFA measures active NE from clinical samples of sputum. A portion of sputum is first diluted in NEATstik® running buffer before transferring to the *Sample pad*. The sputum sample runs along the strip until it reaches the *Conjugate pad*. The conjugate consists of coloured gold nanoparticles conjugated to a NE ProteaseTag, with the latter facilitating capture of active NE through the formation of an irreversible covalent bond within the protease’s active site. For simplicity, the complex of active NE and conjugate will be referred to as *captured NE*. The *Test Line* region of the LFA is used as a trap to immobilise captured NE, whereas the *Control Line* region serves to retain any residual free conjugate and judge that the test has been processed successfully. The remaining fluid that flows beyond the control line is absorbed by the *Absorbing pad*.

As captured NE begins to accumulate at the Test line, a colour change due to densification of the conglomerating gold nanoparticles, proportional to the concentration is produced. For example, in the case the sputum sample has no NE present, the Test Line portion does not change colour. However, for increasing NE concentrations, a more intense or brighter Test Line band is identified. Therefore by measuring the strength of the Test Line colour change, a quantitative estimate of NE concentration can be achieved. A colour change at the Control line should always be present as this denotes that the test has performed correctly.

### B. Imaging of NEATstik Used in the Analysis

**FIGURE 2.**
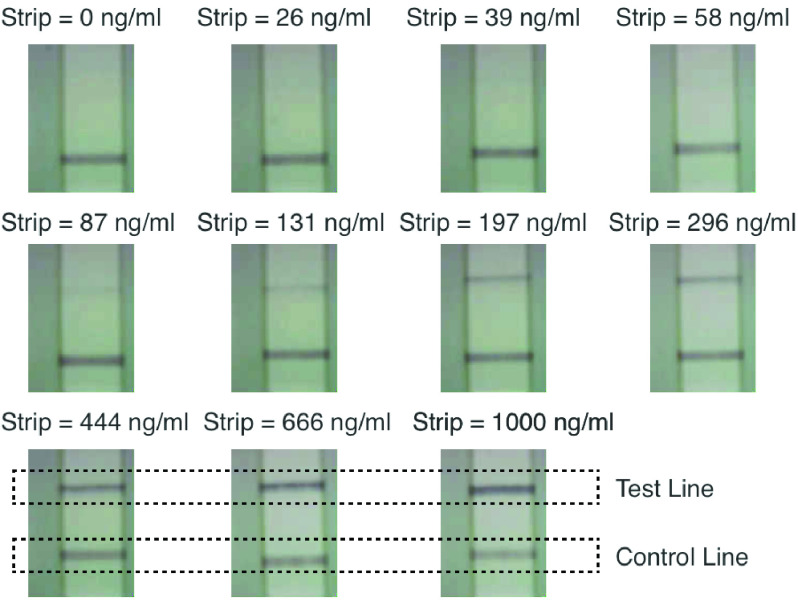
Images taken by the CMOS reader of different NEATstiks with NE concentrations between 0 ng/ml and 1000 ng/ml.

Figure 2 shows grayscale images of NEATstik® LFAs used in the analysis. Each NEATstik® LFA in the image has a different NE strip concentration between 0 ng/ml and 1000 ng/ml. The LFA images were taken using the CMOS camera [15] discussed further in Section III.

Figure 2 shows that when the strip concentration is equal to 0 ng/ml, only the Control Line is present. The Control line is always present for all strip concentrations as a dark red region on the LFA. As the NE strip concentration increases, a second dark region becomes visible above the Control Line. This second region is the Test Line and the intensity increases for higher strip concentrations. By visual analysis, the different strip concentrations in Figure 2 can be classified into different semi-qualitative, but highly subjective, groups:

1) 0 ng/ml to 87 ng/ml: Difficult to determine the presence of a Test Line. This concentration region can be classed as a negative result.
2) 131 ng/ml to 296 ng/ml: Starting to see the formation of a low visibility Test Line. This concentration region can be classed as a low concentration.
3) 444 ng/ml to 1000 ng/ml: A dark high intensity Test Line is visible. This concentration region can be classed as a high concentration.

Using studies carried out by Chalmers *et al.* [14], high NE concentrations using the NEATstik® LFA would correspond to 250 to 300 ng/ml. The visual analysis of the LFA images in Figure 2 shows the formation of a visible Test line for concentration as low as 131 ng/ml, indicating a patient with a high risk of exacerbations. However, the challenge with the visual classification is the NE concentration of the patient sample and the overall change in the trend of the NE concentration is unclear. Therefore any monitoring of the patient’s health is difficult and inaccurate, particularly at the lower concentrations where changes in the Test Line intensity is not clearly visible in the images. The paper aims to develop a suitable NEATstik® reader, similar to other LFA readers [1], to provide a quantitative approach to estimate the patient’s NE concentration. A historical log of the patient’s measured NE concentration can be used to track changes during the treatment period and tailor the medications to improve the patients quality of living.

## III. Methodology

The designed NEATstik® reader can be divided into three parts:

1) CMOS camera design
2) Image analysis approach
3) Data viewing and IoT communications

### A. CMOS Reader Design

CMOS cameras are widely used in several medical diagnostic applications [16]. The technology of the current CMOS camera is constantly refined, resulting in improved image quality, low cost and portability. The low-cost OpenMV H7 camera module is chosen for the reader design presented in this paper [17]. The camera module uses a OV7725 image sensor with the capability of taking $640\times 480$ images, either as 8-bit *Grayscale* or 16-bit *RGB565*, at 75 FPS. The camera module uses a 2.8mm adjustable lens that provides flexibility to constantly produce focused images despite changes to the reader at different stages of the design [17]. The sensor has a standard M12 lens mount allowing the change of lenses for different applications.

The OpenMV H7 also has a micro-controller that can run MicroPython [18]. This allows the OpenMV H7 module to run complex post-processing image analysis for feature extraction and algorithm-based decision making onboard the module and allows the designed system to be a truly portable device that can be operated in any environment. The use of MicroPython also allows the easy integration of Internet of Things (IoT) compatibility and an LCD screen, allowing each reader to be a self-contained diagnostic tool that can be used in a home environment and transmit the results to a secure server to store the results. With the help of data analytics, the historical log of the patient’s health can be used by medical professionals to better tailor the treatment to the patient.

**FIGURE 3.**
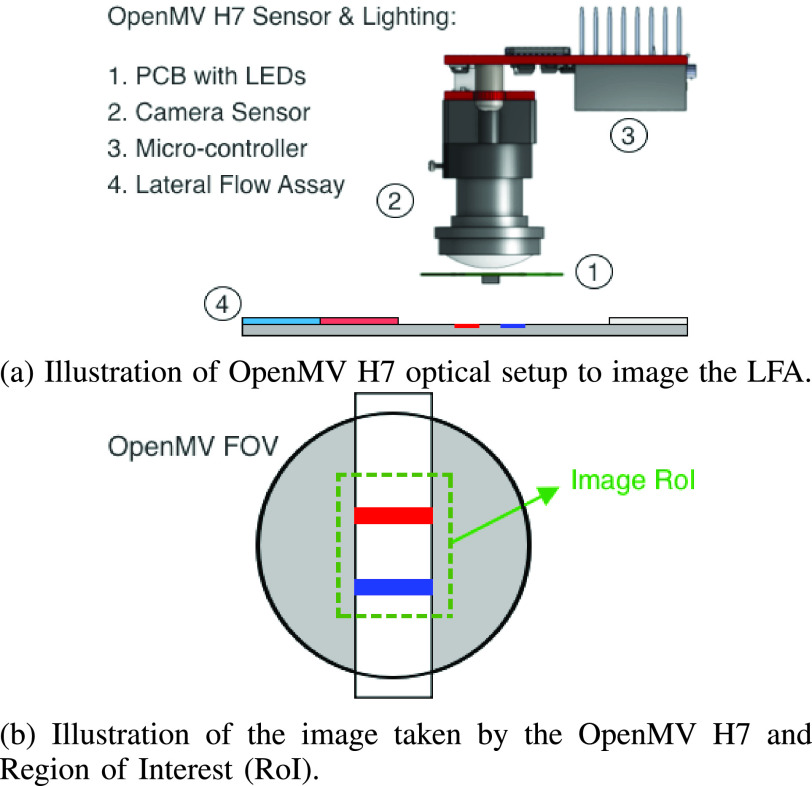
Illustrations of using OpenMV H7 camera used to image Lateral Flow Assays (LFA).

Figure 3 presents an illustration of the designed CMOS reader. The casing used to house the CMOS camera is designed and 3D printed in-house. A PCB containing two LEDs are used to illuminate the NEATstik® LFA strip to provide stable and uniform lighting around the Region of Interest (RoI) of the NEATstik®. The reader can be powered using either a Lithium-ion battery or connecting a micro-USB to the OpenMV H7 camera. The designed CMOS reader had a simplified approach to avoid mechanical and optical complexity. This was possible due to the complexity of the system being shifted to the software side of the image analysis thanks to the advantage offered by the OpenMV H7 camera.

### B. Image Analysis

**FIGURE 4.**
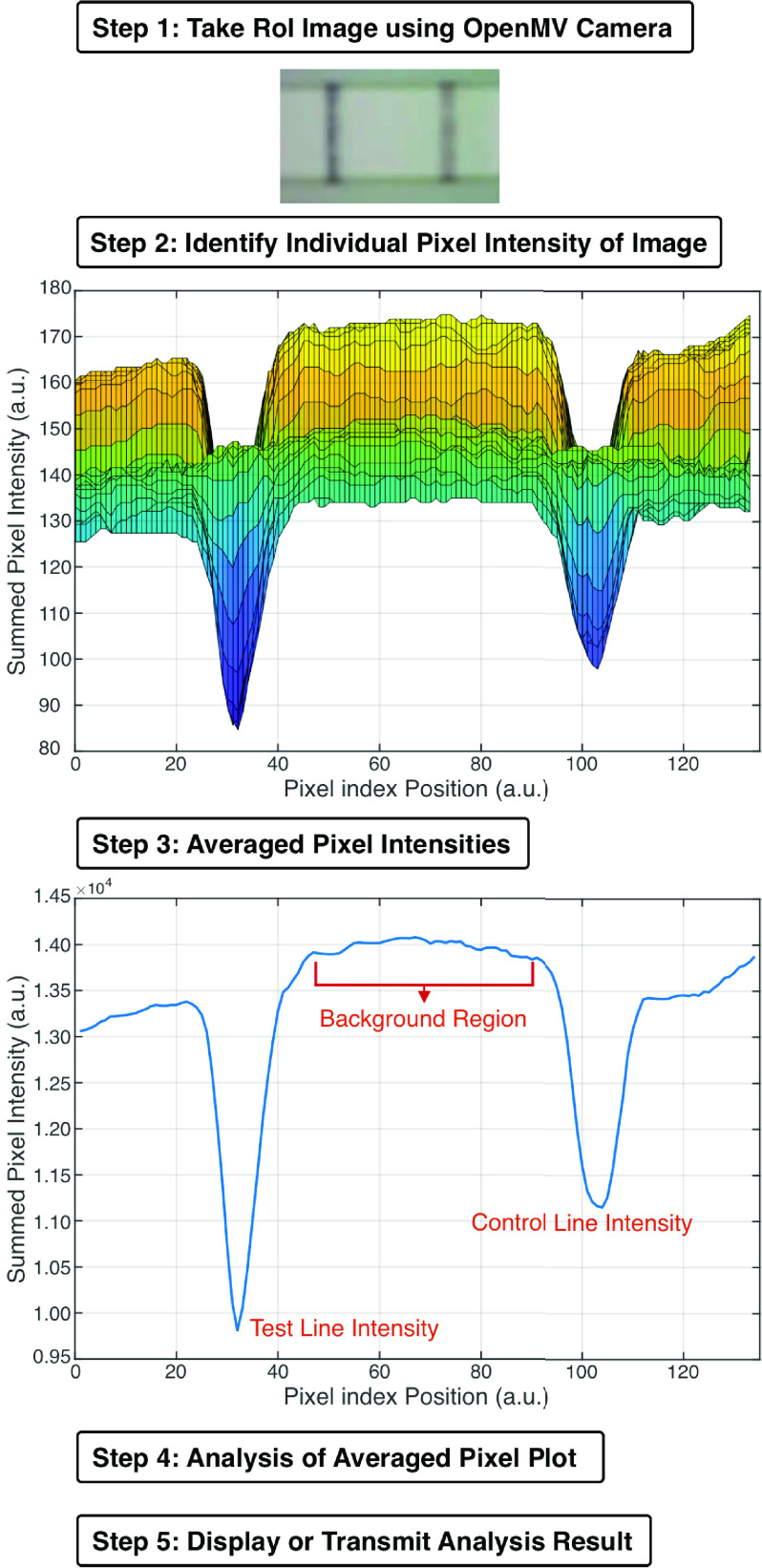
Flowchart with illustration of the image analysis steps to measure different section of the LFA strip.

Figure 4 illustrates the individual steps in the image analysis algorithm carried out on the CMOS reader to estimate the Test and Control Line intensities of the NEATstik®.

In Step 1, the OpenMV camera is used to take a *grayscale image* of the NEATstik® strip and crop the image to only show the RoI that will be analysed. In Step 2, the intensities of individual pixels of the image taken by the OpenMV camera is recorded. In this example, the imaged NEATstik® LFA has a strong visible Test and Control line. The example shows that there is a significant change in the individual pixel intensities, moving from left to right of the NEATstik® due to the Test and Control line.

The plot in Step 2 shows significant variations in the individual pixel values due to either shot noise in the image or background variations. In the case when the Test line is faint, there is greater difficulty to separate the changes to the pixel intensity from these noise sources in the image. Therefore, the averaged pixel intensity is produced in Step 3 to improve the signal-to-noise ratio of the change in pixel information. The plot in Step 3 shows the averaged pixels with two significant dips in the intensity level for the Test line and Control line. The region between the Test line and Control line is called the background region and is used to estimate the average background.

In Step 4, the averaged pixel intensity plot is analysed. Assuming the NEATstik® LFA strip development has worked properly, a Control line will always be produced and present in the image. The onboard algorithm does a scan of the averaged pixel plot in Figure 4 to determine the value and position of the lowest average pixel value of the Control line. By knowing the Control line position, the Test line position can then be estimated. The algorithm places a small window around the expected region of the Test line and performs a second scan. The lowest pixel value and position are used as the Test line value and position. Using the Test and Control line positions, the algorithm defines the background region. The algorithm finds the mean averaged pixel value of the background region and stores that as the mean background.

The output of the algorithm provides the Test-Background, Control-Background and Test-Control Line ratios. The main reason for calculating these ratios between the Test and Control lines with the background is to reduce any common noise, such as from variations in the lighting due to background, and normalise the result to reduce errors in estimating the strip concentration. The Test-Control line ratio is also another way of viewing the results as using the variations in the intensity of both the Test and Control lines could provide researchers with some indication on how the NEATstik® LFA performed at different concentrations.

### C. Image Viewing and Communications

**FIGURE 5.**
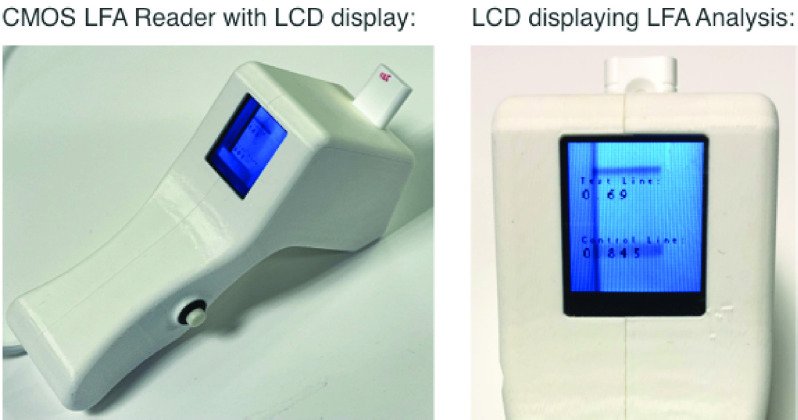
Images showing the designed CMOS LFA reader with the LCD screen showing the LFA strip analysis results of Test and control lines.

Figure 5 shows an image of the CMOS reader. The case was 3D printed using in-house facilities to incorporate the CMOS camera and additional back-end electronics such as LCD screen and IoT boards to present the image analysis results or to send them to a secure server respectively. The case is designed to provide a stable platform for the CMOS camera and control the internal lighting for proper imaging of the NEATstik® LFAs. In Figure 5, the CMOS camera and onboard electronics take and analyse the image and display the Test-Background Ratio and Control-Background ratio on the LCD screen. This allows the device to be operated in a laboratory or clinical environment without the requirement to be connected to a WiFi or Bluetooth device.

**FIGURE 6.**
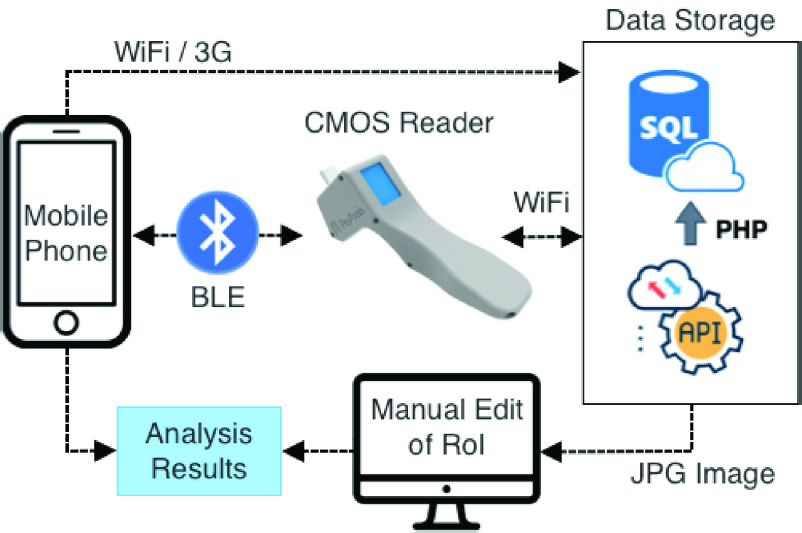
Illustration of image transfer from the reader to smartphone app or web server.

Figure 6 shows the architecture of the IoT version of the reader where both the image and analysis results are transmitted using either a Bluetooth or WiFi board connected to the OpenMV module. For the Bluetooth version, a mobile phone app is used and the results can also be transmitted from the smartphone to a secure server. For the WiFi version, the results are transmitted directly to the secure server. In both IoT approaches, the original image is stored to allow researchers or medical professionals access to the original image of the NEATstik® LFA to test and carry out more complex image analysis methods.

All three methods, LCD and the two IoT methods, were tested and used to analyse the NEATstik® LFAs both for onboard analysis and analysis using a mobile phone and cloud-based system where the researcher or medical professional can control the RoI and analyse the desired section of the images. The results presented in the paper were obtained using the LCD method to fully test the onboard capability of the CMOS reader.

## IV. Results

The 10 NEATstik® NE concentrations shown in Figure 2 were inserted into the reader and the three different ratios produced from the image analysis are logged to determine the trend of the different NE concentrations. For each concentration, 5 different replicas of the LFA strips were used in the analysis. The results are used to produce the mean and standard deviation of estimating the three different ratios.

The results are also compared to another LFA diagnostic tool called the *Lumos Leelu Diagnostics* reader with the same LFA strips. The Lumos system is a commercially available and highly sensitive diagnostic tool used in laboratory environments to aid in the development and testing of LFA strips and has been used in the analysis of LFA in different applications [19]. The Lumos system uses a high-resolution Charged-Coupled Device (CCD) to produce high-quality images for the analysis. The high sensitivity of the Lumos system is capable of measuring small intensity changes in the Test line. Comparing the Lumos system and CMOS system results will help determine the minimum NE concentration that can be detected on the NEATstik® LFA using the CMOS system.

### A. CMOS Reader Analysis Results

**FIGURE 7.**
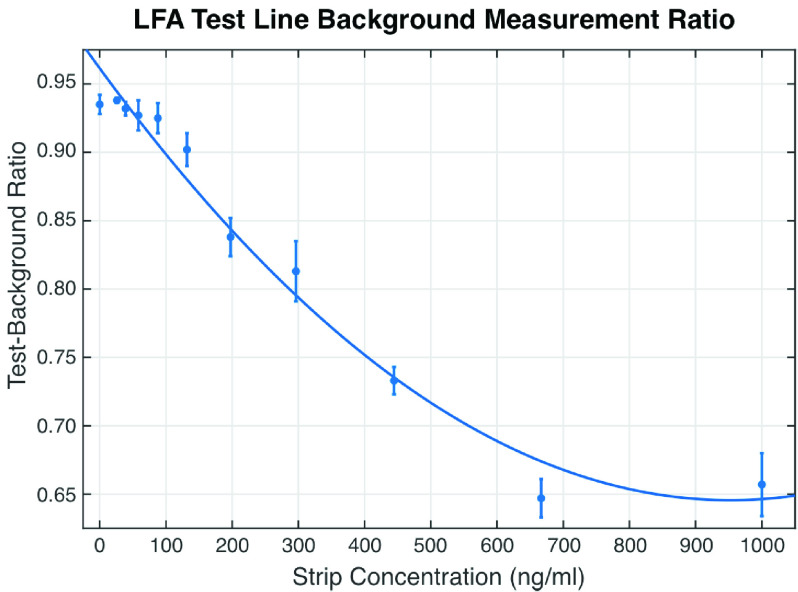
Result showing the change in the Test-Background line ratio for the different NE concentrations.

Figure 7 shows the mean and standard deviation of the Test-Background ratio for each of the NE concentrations. A quadratic trend line is also fitted to the mean Test-Background ratio in Figure 7. Using the result from Figure 7, the Test-Background ratio reduces with a linear relationship with increasing NE concentration up to 600 ng/ml. This indicates that the CMOS reader can read the changes in NE concentration and provide a quantitative estimate. However, for concentrations lower than 100 ng/ml, the standard deviation of Test-Background ratio overlap each other indicating reduced sensitivity of the CMOS reader in estimating the NE concentration in this region.

For concentration above 600 ng/ml, the mean Test-Background ratio starts to plateau but more concentration points are required to further study this region. However, as this paper focuses on the lower concentrations below 400 ng/ml, this effect is not further investigated.

**FIGURE 8.**
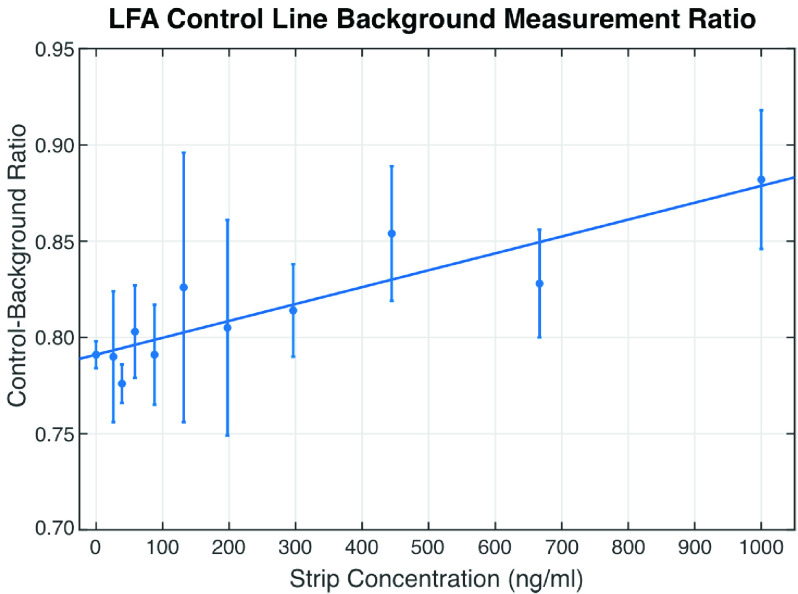
Result showing the change in the Control-Background line ratio for the different NE concentrations.

Figure 8 shows the Control-Background line ratio for the different NE concentrations. The result shows that there are variations in the Control line intensity for different strips of the same strip concentration. These variations may impact the estimation of the strip concentration if the Control line is used as part of the analysis. Further signal processing approach could help reduce these variations and improve the overall analysis results.

**FIGURE 9.**
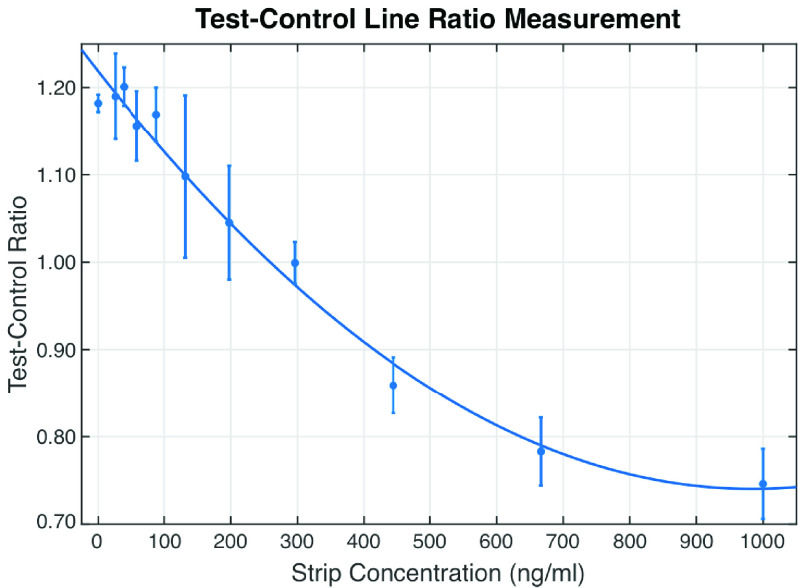
Result showing the change in the Control-Background line ratio for the different NE concentrations.

Figure 9 shows the Test-Control line ratio for the different NE concentrations. Two interesting observations in the result would help in the future development of the CMOS reader. The first is that for concentrations above 300 ng/ml, equivalent to a Test-Control Line ratio of 1, the mean Test-Control Line ratio values better fit the quadratic trend line than the Test-Background ratio in Figure 7. The upper bound range of the NEATstik® analysis also appears to occur at approximately 700 ng/ml, a higher concentration than in Figure 7. However, the second observation shows reduced sensitivity at the lower strip concentrations below 300 ng/ml, where it is difficult to distinguish NE concentrations. This is due to the large variations in the Control line intensity seen in Figure 8. This means the Test-Control Ratio is less sensitive than the using Test-Background Ratio for this NE concentration region.

As discussed in Section II, NE concentrations above 250 ng/ml is considered high using the NEATstik®. The result presented in Figure 7 shows that the combination of using a NEATstik® LFA with a CMOS camera reader produces a quantitative trend and Figure 7 can be used to estimate the unknown NE concentration in a patient’s sputum with the desired sensitivity to diagnose a patient with a high risk of exacerbation.

### B. Comparison Between CMOS Reader and LUMOS Reader

**FIGURE 10.**
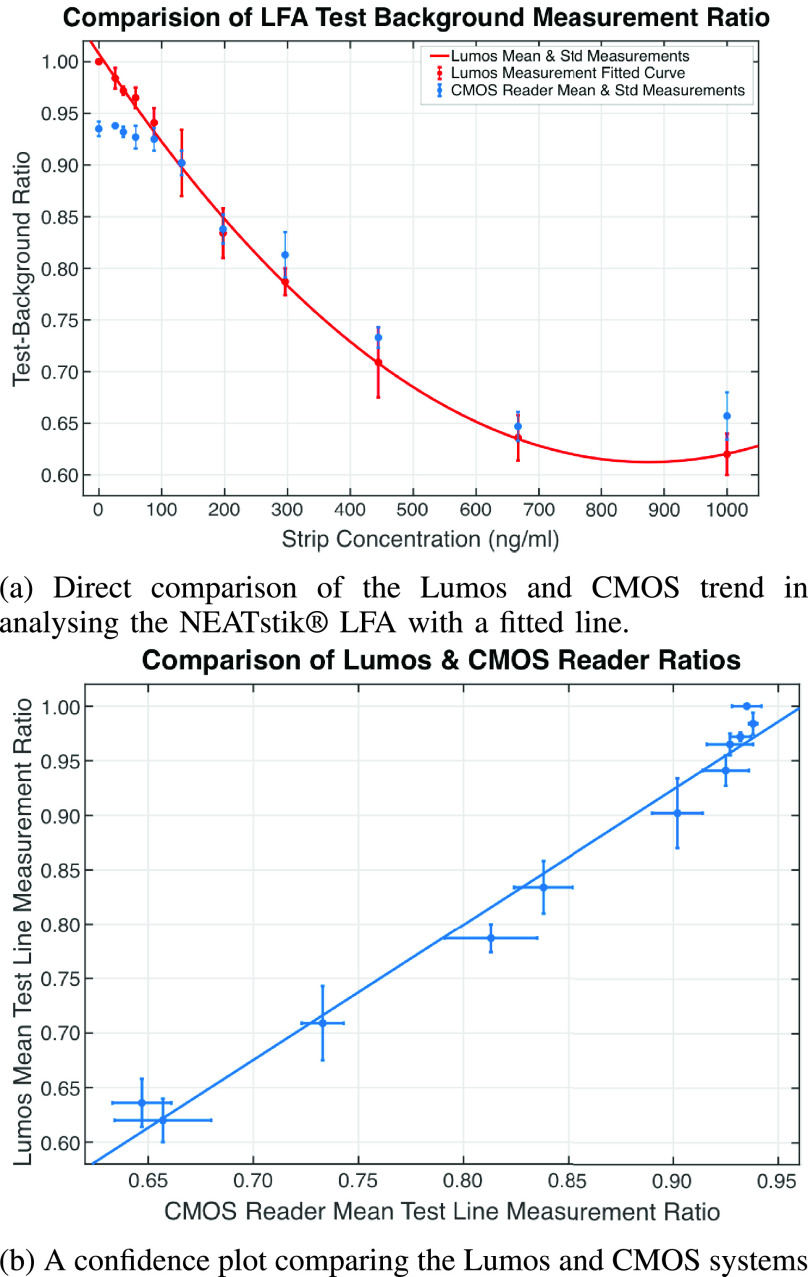
Comparing the NEATstik® LFA analysis using both the CMOS and Lumos LFA readers.

Figure 10 shows the comparison of the Test-Background Ratio between the CMOS reader, from Figure 7, with the Lumos system. Figure 10(a) shows the direct comparison of the Test-Background Ratio of the two systems for different NE concentrations. Figure 10(b) shows a confidence plot that compares the differences and variations in the measurements for the same NE concentrations.

Figure 10(a) shows the CMOS system matches the Lumos system for NE concentrations above 100 ng/ml. Figure 10(b) shows the error bars for both CMOS and Lumos systems are comparable for NE concentrations above 100 ng/ml. Both these results show that the CMOS system is capable of matching the performance of the Lumos system. However, Figure 10(a) also shows that for NE concentrations below 100 ng/ml, the CMOS reader measurements trend flattens but the Lumos system is still able to measure the small changes in the NEATstik® LFA Test line intensity. This is due to the higher quality imaging by the Lumos system compared to the CMOS reader and hence can produce a much better signal-to-noise ratio when analysing the images.

The results from Figure 10 highlights two key factors. The first is the NEATstik® LFA, together with a sensitive laboratory-based diagnostic tool such as the Lumos system, can produce a calibration curve. The second factor is the CMOS system can match the Lumos system but struggles to distinguish low Test line intensity changes for NE concentrations between 0 ng/ml and 100 ng/ml. This lack of sensitivity of the CMOS system is due to the lower resolution images for analysis compared to the Lumos system and hence the reduced signal-to-noise ratio.

Even though the CMOS system presented in this paper is less sensitive than the Lumos system, the CMOS camera approach allows several advantages for a PoC application. The CMOS system is a low-cost system while the Lumos system is an expensive laboratory calibrated system. The modular layout of the CMOS system allows continual development and improvements to achieve higher resolution images. Using a narrower field of view lens and replacing the current OV7725 image sensor can better focus the image around the RoI and improve image resolution and sensitivity. The CMOS camera allows the onboard implementation of up-to-date image analysis and feature extraction algorithms, with the ability to transmit the images and results using IoT capabilities. In comparison, the images from the Lumos system have to be transferred onto a computer to perform the image analysis with software such as MATLAB or ImageJ.

## V. Future Work

The results presented in this paper demonstrate the feasibility of using a CMOS lateral flow reader to analyse NEATstik® LFAs and assist in the diagnosis of impending respiratory exacerbations. The results also show the sensitivity of future iterations of the CMOS reader can be improved to better estimate lower NE concentrations.

Future iterations of the reader will focus on two main areas. The first will be improving image quality around the RoI of the LFA by using optical lenses with narrower FOV and higher resolution CMOS sensors. This will improve the number of useful pixel information available for the RoI during the analysis. The second area to focus on will be improving the analysis approach for better feature extraction of the RoI. This includes using pre-analysis methods to improve image quality by changing the contrast in the image and removal of unwanted background portions in the image. More robust feature extraction analysis methods will be investigated, including integrating the previous work related to using neural network [4] to analyse the LFA images to help to identify smaller changes in the Test line intensity.

The updated CMOS reader will also be used to analyse large samples of NEATstiks LFAs for different NE concentrations. This is to determine the effectiveness of the overall system for PoC application in a medical or clinical environment.

## VI. Conclusion

This paper investigated a PoC approach to determine the NE concentrations from a patient’s sputum related to respiratory illness using a lateral flow assay called a NEATstik®. A CMOS camera is integrated into the reader design to perform onboard image analysis of the NEATstik® to determine the NE concentration in the sputum. The designed LFA CMOS reader presented in this paper can also be adapted to image and analyse LFAs for with other biomarkers for detection of different medical health issues such as heart failure or cancer.

The results presented in the paper compared the CMOS system to the Lumos diagnostic system, a sensitive laboratory lateral flow reader.The analysis results shows both systems can provide quantitative measurements of the NEATstik® LFAs. he calibration curve produced by the CMOS reader is comparable to the Lumos diagnostic system for concentrations above 100 ng/ml. Despite the lack of sensitivity at concentrations below 100 ng/ml compared to the Lumos diagnostic system, the CMOS reader can estimate the NE concentration from a patient’s sputum and determine if the patient is at high risk from respiratory exacerbations.

Despite the current limitations of the CMOS reader compared to the Lumos system, future developments of the CMOS reader will improve the image quality and hence, the sensitivity at the lower NE concentration. With the implementation of IoT capability and the ability to implement onboard algorithms, the aim is to develop a portable and easy to use PoC diagnostic tool to continually monitor patient’s health and improve quality of living.
